# The role of deviant leisure tendencies and loneliness in gambling craving

**DOI:** 10.3389/fpubh.2026.1831870

**Published:** 2026-05-28

**Authors:** Sinan Erdem Satılmış, Ozan Yılmaz

**Affiliations:** 1 Department of Recreation, Faculty of Sport Science, Yalova University, Yalova, Türkiye; 2 Department of Recreation, Faculty of Sport Science, Istanbul Yeni Yuzyil University, Istanbul, Türkiye

**Keywords:** deviant leisure, gambling, loneliness, tendencies, university students

## Abstract

The aim of this study is to examine the relationships between gambling craving, deviant leisure tendencies, and loneliness among university students. The study was conducted using a cross-sectional correlational design with a sample of 521 students from different faculties. Data were collected using the Gambling Craving Scale (GCS), the Adult Deviant Leisure Tendency Scale (ADLTS), the UCLA Loneliness Scale (ULS-8), and analyzed using *t*-tests, Pearson correlation, and hierarchical regression analysis. Correlation analysis indicated that gambling craving was strongly correlated with deviant leisure tendencies, whereas loneliness showed a weaker but statistically significant correlation. When examined within a hierarchical regression model controlling for demographic and behavioral variables, deviant leisure tendencies demonstrated a substantially stronger association with gambling craving compared to loneliness. In addition, group comparisons revealed that individuals reporting passive leisure engagement and substance use exhibited higher levels of gambling craving. Overall, the findings suggest that gambling craving may be more appropriately understood within the broader context of risk-oriented leisure patterns. However, these results should be interpreted cautiously given the cross-sectional design and reliance on self-report measures, and they do not imply causal relationships. Accordingly, practical implications should be considered as preliminary.

## Introduction

Gambling has increasingly become a common practice among university students in recent years and is often experienced as part of entertainment, socialization, and leisure (1, 2). Particularly during emerging adulthood increased freedom, expanded leisure opportunities and greater digital access create conditions that make gambling more visible and accessible in everyday life (3). Many studies report that a considerable proportion of university students have experienced gambling at some point in their lives and that gambling is often perceived as a harmless form of entertainment or a routine leisure activity (4).

In the literature gambling has frequently been examined through individual characteristics such as psychopathology, impulsivity, risk taking, and sensation seeking (5–7). Studies conducted with university student samples indicate that gambling tends to cluster with alcohol and substance use, academic problems, and other risky behaviors (8–10). These approaches often position gambling primarily as an individual problem and address the functioning of the behavior within its social context at a secondary level. When individuals fail to meet their psychosocial needs or lose their capacity to generate meaning this sphere may create a ground for risky and non-normative behaviors. The literature particularly shows that unstructured leisure may foster problematic behavioral patterns through processes such as boredom, sensation seeking, and loss of control (11, 12).

While the broader conceptual framework emphasizes the role of social and contextual factors in shaping gambling-related behaviors, the present study focuses specifically on individual-level leisure dispositions and their association with gambling craving. In this sense, the study does not directly measure contextual variables but aims to provide an initial empirical step toward understanding these processes at the individual level.

Within this framework, gambling-related engagement is increasingly evaluated not only as a form of entertainment or a recreational activity but also as part of risky and deviant leisure patterns (13). Previous studies indicate that gambling frequently co-occurs with substance use, impulsivity, and other deviant behaviors, and is positioned within a broader cluster of deviant behavioral patterns (14, 15, 30). This perspective suggests that gambling may be approached not as an isolated behavior but as a leisure orientation characterized by sensation seeking, risk taking, and escape motivations. Accordingly, deviant leisure behaviors emphasize norm violations and the potential for individual and social harm, suggesting that certain leisure activities may gradually become intertwined with risky behavioral patterns over time (16, 17). While the present study draws on this theoretical perspective, it focuses specifically on one dimension of the deviant leisure framework, namely individual-level transgressive leisure tendencies. The broader structural and cultural processes emphasized by Raymen and Smith are not directly examined in the current study. Therefore, the findings should be interpreted within this narrower scope rather than as a comprehensive test of the deviant leisure framework.

Consistent with this narrower operationalization, it is also important to consider the potential conceptual proximity between the key variables examined in the study. While deviant leisure tendencies and gambling craving are conceptually related constructs, their empirical association may partly reflect a degree of conceptual and measurement overlap. Deviant leisure tendencies capture a broader orientation toward norm-violating and risk-seeking leisure preferences, whereas gambling craving reflects a more specific motivational tendency toward gambling-related engagement. Therefore, the relationship between these variables should not be interpreted as purely explanatory, but rather as indicative of overlapping behavioral and motivational patterns within the leisure context. Recognizing this potential overlap is important in order to avoid tautological interpretations and to situate the findings within a more cautious conceptual framework.

In addition to deviant leisure tendencies loneliness has emerged as an important psychosocial variable associated with gambling (18). Loneliness may trigger negative affect avoidance tendencies and compensatory behaviors together with the perception of inadequacy in an individual's social relationships. In this context gambling may become a tool that provides temporary pleasure distraction and a sense of pseudo social interaction for individuals experiencing loneliness (19). Research indicates that loneliness is associated with gambling however this relationship is often shaped through interactions with other risk factors rather than acting as a single determining factor (20, 21). These findings suggest that the role of loneliness in gambling should be evaluated not only in terms of individual experiences but also together with leisure patterns and the dynamics of social interaction. In this study, loneliness is conceptualized as a background psychosocial factor that may be associated with gambling craving rather than a direct or primary explanatory variable. Given the cross-sectional nature of the data, the study does not test mediation or moderation mechanisms but instead focuses on examining the relative strength of associations between loneliness and gambling craving compared to deviant leisure tendencies. This distinction is important in order to align the theoretical framing with the empirical design.

Although the relationships between gambling craving, deviant leisure tendencies, and loneliness have been examined separately in the literature, studies that address these variables together within a coherent leisure-based framework remain limited. More importantly, the existing literature often does not sufficiently clarify how these constructs are conceptually related or how their associations should be interpreted.

In this context, the present study aims to examine the relationships between gambling craving, deviant leisure tendencies, and loneliness within a correlational framework. Rather than proposing a causal model, the study focuses on comparing the relative strength of these associations and situating them within the broader context of leisure behavior. By doing so, the study seeks to contribute to the literature by offering a more conceptually cautious and analytically grounded interpretation of these relationships.

Based on the conceptual framework and empirical findings in the literature, the following hypotheses were formulated:

H1: Deviant leisure tendencies will be positively associated with gambling craving among university students.

H2: Loneliness will be positively associated with gambling craving, but this relationship will be weaker compared to deviant leisure tendencies.

H3: Individuals who engage in passive leisure activities and report substance use will exhibit higher levels of gambling craving compared to those with active leisure participation and no substance use.

## Materials and methods

### Research model

This study employed a cross-sectional correlational survey design to examine the relationships between deviant leisure tendencies, loneliness, and gambling craving among university students. This design allows for the investigation of associations between variables without manipulation and is appropriate for identifying the direction and strength of relationships within a given sample.

### Sample group

The study sample was determined using a convenience sampling method, which is a non-probability sampling technique. A total of 521 university students from Yalova University and Istanbul Yeni Yüzyıl University voluntarily participated in the study. The characteristics of the sample group are presented in Table 1.

**Table 1:** Characteristics of the sample group.

Variables	Subgroups	Frequency	%	Mean	S.D.	Total
Gender	Female	217	41,7			521
	Male	304	58.3			
Age				21.10	3.71	
GPA				2.73	0.52	
Weekly leisure duration				32.03	17.07	
Weekly screen time				16.72	4.44	
Leisure type	Active	338	64.9			
	Passive	183	35.1			
Cigarettes/Tobacco and alcohol use	Yes	298	57.2			
	No	223	42.8			

The sample consisted of students from different faculties and academic levels. University students were considered an appropriate population due to characteristics such as increased autonomy, unstructured leisure time, and relatively easier access to risk-related behaviors.

Given the use of convenience sampling from two universities, the findings should be interpreted with caution and are not intended to be generalized to all university student populations.

### Measures

In the study, three standardized measurement tools were used along with a Personal Information Form prepared by the researchers. The selection of these scales was based on their theoretical relevance, prior use in university student samples and established validity and reliability evidence. In addition to previously reported psychometric properties, the internal consistency coefficients of all scales were recalculated for the current sample and are reported in the findings section.

### Personal information form

The Personal Information Form included items on gender, leisure type, substance use, GPA, weekly leisure duration, and weekly screen time. Leisure type (active vs. passive) was assessed based on participants' self-reported predominant way of spending leisure with physically, socially, or participatory activities categorized as active and screen-based or sedentary activities categorized as passive. Weekly screen time and weekly leisure duration were measured through self-report items, with participants indicating the approximate number of hours per week spent on these activities. These variables were treated as continuous in the analyses. Substance use was coded as a dichotomous variable (yes/no) based on participants' self-reports regarding cigarette/tobacco, alcohol, and similar substance-related behaviors. This variable was intentionally retained to capture a broader risk-related behavioral orientation rather than isolated substance use patterns. GPA was obtained through self-report and treated as a continuous variable. In Turkish higher education institutions, GPA is calculated on a standardized 4.00 scale, which ensures comparability across universities.

### Gambling Craving Scale (GCS)

The Gambling Craving Scale developed by Young and Wohl (22) was used to assess the level of gambling craving. The scale evaluates the cognitive and emotional processes associated with the urge to gamble, including thoughts related to the desirability of gambling, perceived need, anticipated emotional relief, and difficulties in self-control. The Turkish validity and reliability study was conducted by Buran et al. (23). The scale consists of 8 items with a single-factor structure and is rated on a 7-point Likert scale. Higher scores indicate higher levels of gambling craving.

### Adult Deviant Leisure Tendency Scale (ADLTS)

The Adult Deviant Leisure Tendency Scale developed by Bedir et al. (24) was used to assess individuals' tendencies toward leisure activities involving risk and norm violations. The scale has a single-factor structure and consists of 5 items measuring orientations toward sensation seeking, boundary testing, and engagement in non-normative leisure behaviors. Specifically, the items reflect preferences for intense and unrestrained leisure experiences, association with potentially risky social environments, engagement in substance-related leisure activities, participation in unconventional or norm-violating experiences and easy sexual encounters within leisure contexts.

Although the ADLTS provides a concise measure, its limited number of items captures a relatively narrow range of risk-oriented leisure tendencies. Therefore, it reflects a general disposition toward non-normative and risk-seeking leisure rather than a specific behavioral outcome. In contrast, gambling craving represents a more specific cognitive and emotional tendency toward gambling-related engagement.

While these constructs share a common underlying dimension related to risk and sensation seeking, they operate at different levels of abstraction. Accordingly, their association should be interpreted as an empirical relationship between a general leisure orientation and a specific motivational tendency rather than a definitional overlap. Nevertheless, this conceptual proximity should be considered when interpreting the strength of the observed relationship.

### UCLA Loneliness Scale (ULS-8)

Loneliness levels were measured using the 8-item short form of the UCLA Loneliness Scale developed by Hays and DiMatteo (25), based on the original scale by Russell et al. (26). The Turkish adaptation was conducted by Dogan et al. (27). The scale has a single-factor structure and is rated on a 4-point Likert scale. Higher scores indicate higher levels of loneliness. Items 3 and 6 are reverse coded.

### Data collection

Data were collected using a mixed-mode approach, including both online surveys (Google Forms) and face to face questionnaires. Participants were informed about the purpose of the study, confidentiality, and voluntariness, and informed consent was obtained prior to participation. Specifically, of the final sample included in the analyses (*N* = 521), 413 participants (79.3%) completed the survey online, whereas 108 participants (20.7%) responded via face-to-face administration.

Although mixed mode data collection may introduce potential mode effects, this issue was subsequently tested and statistically controlled in the regression analyses.

### Data analysis

The data were analyzed using the SPSS 23.0 statistical package program. Descriptive statistics (frequency and percentage) were used to summarize participant characteristics. The internal consistency of the scales was evaluated using Cronbach's alpha coefficients.

Normality assumptions were assessed through skewness and kurtosis values. Pearson correlation analysis was conducted to examine the relationships between variables, and hierarchical multiple regression analysis was used to assess the extent to which deviant leisure tendencies and loneliness are associated with gambling craving while controlling for demographic and behavioral variables. The level of statistical significance was set at 0.05 for all analyses.

In addition, regression assumptions were examined prior to the analysis. Visual inspection of residual plots indicated no serious violations of linearity; however, a slight pattern suggested a potential minor deviation from homoscedasticity. The normal P–P plot indicated that residuals were approximately normally distributed, although minor deviations were observed in the middle range, likely due to the nature of Likert-type data.

Collinearity diagnostics indicated no evidence of multicollinearity among the predictors (all VIFs < 2). Influence diagnostics further showed that no individual observation exerted a disproportionate effect on the model (maximum Cook's Distance = 0.062).

### Findings

When Table 2 is examined the mean score obtained by the participants from the Gambling Craving Scale (GCS) was determined as 2.54, the mean score obtained from the Adult Deviant Leisure Tendency Scale (ADLTS) was 2.50 and the mean score obtained from the UCLA Loneliness Scale (ULS-8) was 1.79. When the internal consistency coefficients of the scales were examined the Cronbach's Alpha values were found to be 0.957 for the GCS, 0.899 for the ADLTS, and 0.769 for the ULS-8. These values indicate that the reliability levels of the scales are acceptable and high. When the distribution characteristics of the sample group were examined the skewness and kurtosis values being within the range of ±1.5 indicate that the data show a distribution close to normal (28). Accordingly the obtained data were considered appropriate for parametric analyses.

**Table 2:** Descriptive statistics of study variables.

Variables	*N* of items	*n*	Mean	S.D.	Skewness	Kurtosis	Cronbach's alpha
Gambling Craving Scale (GCS)	8	521	2.54	1.65	0.613	−0.943	0.957
Adult Deviant Leisure Tendency Scale (ADLTS)	5	2.68	2.50	1.07	0.104	−1.200	0.899
UCLA Loneliness Scale (ULS-8)	8		1.79	0.49	0.700	1.333	0.769

When Table 3 is examined, the mean scores of the Gambling Craving Scale (GCS) and the Adult Deviant Leisure Tendency Scale (ADLTS) were found to differ significantly according to gender (*p* < 0.001). The mean scores for male participants were higher than those for female participants on both scales. In contrast, the mean scores of the UCLA Loneliness Scale (ULS-8) did not differ significantly according to gender (*p* = 0.393 > 0.05).

**Table 3:** Independent samples *t*-test by gender.

Variables	Gender	*n*	Mean	S.D.	*t*	*df*	*p*	*d*
GCS	Female	217	1.95	1.34	−7.570	514.762	0.001^***^	0.67
	Male	304	2.97	1.72				
ADLTS	Female	217	2.10	0.93	−7.699	501.723	0.001^***^	0.69
	Male	304	2.79	1.08				
ULS-8	Female	217	1.77	0.48	−0.854	519	0.393	0.08
	Male	304	1.81	0.49				

^***^p < 0.001.

Although statistically significant differences were observed for GCS and ADLTS, the effect sizes indicated moderate effects (*d* = 0.67 and *d* = 0.69, respectively). In contrast, no significant difference was found for loneliness, and the effect size was negligible (*d* = 0.08).

When Table 4 is examined, the mean scores of the Gambling Craving Scale (GCS), Adult Deviant Leisure Tendency Scale (ADLTS), and UCLA Loneliness Scale (ULS-8) were found to differ significantly according to the way participants engage in leisure (*p* < 0.001). In terms of GCS, the mean score of participants in the passive participation group (*x* = 3.46) was significantly higher than that of participants in the active participation group (*x* = 2.05). Similarly, the mean ADLTS scores were higher in the passive participation group (*x* = 3.00) compared to the active participation group (*x* = 2.23). In terms of ULS-8, the mean loneliness scores of participants in the passive participation group (*x* = 1.89) were significantly higher than those of participants in the active participation group (*x* = 1.74). These findings suggest that passive forms of leisure participation are associated with higher levels of gambling craving, deviant leisure tendencies, and loneliness compared to active forms of participation.

**Table 4:** Independent samples *t*-test by leisure type.

Variables	Leisure type	*n*	Mean	S.D.	*t*	*df*	*p*	*d*
GCS	Active	338	2.05	1.46	−9.960	346.966	0.001^***^	1.07
	Passive	183	3.46	1.59				
ADLTS	Active	338	2.23	1.00	−8.242	519	0.001^***^	0.72
	Passive	183	3.00	1.03				
ULS-8	Active	338	1.74	0.53	−3.443	480.595	0.001^***^	0.31
	Passive	183	1.89	0.38				

^***^p < 0.001.

The effect size for gambling craving was large (*d* = 1.07), indicating a substantial difference between groups. For deviant leisure tendencies, the effect size was moderate to large (*d* = 0.72), while loneliness showed a smaller but still meaningful effect (*d* = 0.31).

When Table 5 is examined, the mean scores of the Gambling Craving Scale (GCS) and the Adult Deviant Leisure Tendency Scale (ADLTS) were found to differ significantly according to the use of cigarettes/tobacco, alcohol, and similar products (*p* < 0.001). The mean GCS score of participants who reported using these substances (*x* = 3.21) was significantly higher than that of participants who reported not using them (*x* = 1.65). Similarly, the mean ADLTS scores were higher among participants who reported using these substances (*x* = 2.95) compared to those who did not (*x* = 1.90). In contrast, loneliness levels did not show a significant difference according to substance use (*p* > 0.05). The mean loneliness scores of participants who reported using these substances (*x* = 1.79) and those who reported not using them (*x* = 1.80) were found to be similar. These findings suggest that substance use is associated with higher levels of gambling craving and deviant leisure tendencies, whereas no meaningful difference was observed in loneliness levels between the groups.

**Table 5:** Independent samples *t*-test by use of cigarettes/tobacco alcohol and similar products.

Variables	Cigarettes/Tobacco alcohol	*n*	Mean	S.D.	*t*	*df*	*p*	*d*
GCS	Yes	298	3.21	1.60	12.520	518.350	0.001^***^	1.10
	No	223	1.65	1.24				
ADLTS	Yes	298	2.95	0.96	12.422	519	0.001^***^	1.09
	No	223	1.90	0.92				
ULS-8	Yes	298	1.79	0.41	−0.057	383.569	0.954	0.01
	No	223	1.80	0.58				

^*^In this table, cigarette, alcohol, and similar substance use behaviors were combined into a single dichotomous variable to represent a broader risk-related behavioral orientation. ^*^p < 0.05, ^**^p < 0.01, ^***^p < 0.001.

The effect sizes for GCS and ADLTS were large (*d* = 1.10 and *d* = 1.09), indicating substantial practical differences between the groups. In contrast, no statistically significant difference was observed for loneliness, and the effect size was negligible (*d* = 0.01).

Findings presented in Table 6 indicate a positive and strong relationship between the Gambling Craving Scale (GCS) and the Adult Deviant Leisure Tendency Scale (ADLTS) (*r* = 0.760). The strong correlation observed between the Adult Deviant Leisure Tendency Scale (ADLTS) and the Gambling Craving Scale (GCS) (*r* = 0.760) indicates a substantial degree of shared variance between the constructs. This pattern suggests that the two measures may capture closely related aspects of a broader risk-oriented behavioral domain rather than entirely distinct phenomena. A positive but low-level relationship was found between the UCLA Loneliness Scale (ULS-8) and GCS (*r* = 0.184) and ADLTS (*r* = 0.168), indicating that loneliness shows more limited associations with these constructs.

**Table 6:** Correlation analysis results between GCS, ADLTS, and ULS-8 and GPA, weekly leisure duration, and weekly screen time.

Variables		GCS	ADLTS	ULS-8	GPA	Weekly leisure duration	Weekly screen time
GCS	*r*	1					
	*p*						
ADLTS	*r*	0.760^***^	1				
	*p*	0.001					
ULS-8	*r*	0.184^***^	0.168^***^	1			
	*p*	0.001	0.001				
GPA	*r*	−0.352^***^	−0.347^***^	−0.110^**^	1		
	*p*	0.001	0.001	0.012			
Weekly leisure duration	*r*	0.472^***^	0.499^***^	0.079	−0.372^***^	1	
	*p*	0.001	0.001	0.073	0.001		
Weekly screen time	*r*	0.376^***^	0.358^***^	0.044	−0.226^***^	0.391^***^	1
	*p*	0.001	0.001	0.315	0.001	0.001	

^**^p < 0.01, ^***^p < 0.001.

Negative and low-level relationships were found between GPA and GCS (*r* = −0.352) and ADLTS (*r* = −0.347), suggesting that higher academic achievement is associated with lower levels of gambling craving and deviant leisure tendencies. In addition, a negative and low-level relationship was observed between GPA and ULS-8 (*r* = −0.110), indicating that higher academic achievement is associated with lower levels of loneliness.

A positive and moderate relationship was identified between weekly leisure duration and GCS (*r* = 0.472) and ADLTS (*r* = 0.499). However, no significant relationship was found between weekly leisure duration and ULS-8. Finally, a positive and low-level relationship was found between weekly screen time and GCS (*r* = 0.376) and ADLTS (*r* = 0.358), while no significant relationship was observed with ULS-8. Overall, the findings indicate that longer leisure duration and higher digital screen time are associated with higher levels of gambling craving and deviant leisure tendencies.

A hierarchical multiple regression analysis was conducted to examine the extent to which deviant leisure tendencies and loneliness are associated with gambling craving after controlling for demographic and behavioral variables. The hierarchical regression analysis results are presented in Table 7. In the first model, gender, age, GPA, leisure type, weekly leisure duration, substance use, weekly screen time, and data collection mode were entered as control variables. This model was statistically significant (*F* = 49.766, *p* < 0.001) and explained 43.7% of the variance in gambling craving (R^2^ = 0.437).

**Table 7:** Hierarchical regression analysis predicting gambling craving.

Variables	Model 1 β	Model 2 β
Gender	0.204^***^	0.081^**^
Age	−0.31	0.005
GPA	−0.108^**^	−0.041
Leisure type	0.171^***^	0.113^***^
Weekly leisure duration	0.151^***^	0.022
Cigarettes/Tobacco and alcohol use	0.256^***^	0.102^**^
Weekly screen time	0.165^***^	0.087^**^
Data collection mode (face to face or online)	−0.080	−0.057
ADLTS	—	0.570^***^
ULS-8	—	0.064^*^
*R^2^*	0.437	0.634
Δ*R^2^*	—	0.197
*F*	49.766^*^	88.495^*^
Δ*F*	—	137.371^*^

^*^p < 0.05, ^**^p < 0.01, ^***^p < 0.001.

In the second model, ADLTS and ULS-8 were added to the analysis. The overall model remained significant (*F* = 88.495, *p* < 0.001), and the explained variance increased to 63.4% (*R*^2^ = 0.634), indicating a substantial increase in explanatory power (Δ*R*^2^ = 0.197, *p* < 0.001).

After controlling for all variables in Model 1, deviant leisure tendencies showed a strong positive association with gambling craving (β = 0.570, *p* < 0.001), whereas loneliness demonstrated a weaker but statistically significant association (β = 0.064, *p* = 0.021). These findings indicate that ADLTS contributes substantial additional variance beyond the control variables, while ULS-8 plays a more limited role within the model.

In addition, data collection mode (0 = face to face, 1 = online) did not emerge as a statistically significant predictor of gambling craving in either Model 1 (β = −0.080, *p* = 0.058) or Model 2 (β = −0.057, *p* = 0.096). The non-significant effect of data collection mode suggests that the observed relationships are unlikely to be driven by differences between online and face-to-face responses.

## Discussion and conclusion

Given the cross-sectional and self-report nature of the study, all interpretations are strictly limited to associations rather than causal relationships. The findings indicate that gambling craving is strongly associated with deviant leisure tendencies, whereas loneliness plays a more limited but statistically significant role. Importantly, the hierarchical regression results demonstrate that this strong association persists even after controlling for multiple demographic and behavioral variables. This strengthens the interpretation that the observed relationship is not solely attributable to shared variance with commonly associated factors such as substance use, leisure patterns, or screen time, and supports the robustness of the association within a multivariate framework.

The strength of the association between deviant leisure tendencies and gambling craving warrants careful interpretation. Rather than indicating a directional or explanatory relationship, the magnitude of this association suggests a substantial degree of conceptual and empirical overlap between the constructs. In this sense, gambling craving may be more appropriately interpreted as a specific cognitive–behavioral expression within a broader constellation of risk-oriented leisure tendencies. This interpretation does not imply a causal hierarchy between the constructs; rather, it reflects the possibility that both measures capture closely related dimensions within a shared behavioral domain characterized by sensation seeking, norm deviation and risk engagement.

These results suggest that evaluating gambling craving solely as an indicator of individual impulsivity or psychopathology may be insufficient, and that it may be more appropriately considered within the broader context of risky leisure orientations. Consistent with this perspective, the hierarchical regression results indicate that deviant leisure tendencies remain strongly associated with gambling craving (β = 0.570, *p* < 0.001) even after controlling for multiple demographic and behavioral variables. This pattern further supports the interpretation that the relationship reflects a robust empirical link within a shared behavioral domain rather than a simple bivariate association.

This finding may be interpreted in light of the conceptual framework of deviant leisure theory. Raymen and Smith (13, 17) suggest that in late modern societies, certain leisure practices may become increasingly oriented toward pleasure consumption and risk, potentially giving rise to non-normative behavioral patterns. Within this context, gambling may function as a source of excitement, identity construction, and escape from the routines of everyday life. Similarly, Franklin-Reible (16) notes that some leisure activities may offer pleasurable experiences while simultaneously carrying the potential for norm violations at the societal level. However, the strong association identified between deviant leisure tendencies and gambling craving in the present study (*r* = 0.760) should be interpreted with caution. This relationship may partly reflect the conceptual proximity between the constructs, as both are related to broader dimensions such as risk orientation and sensation seeking. Therefore, rather than indicating a direct explanatory relationship, the findings may be better understood as reflecting a strong empirical association within a shared conceptual domain.

The literature frequently indicates that gambling tends to co-occur with substance use, impulsivity, and other risky behaviors (14, 15). In the present study, the significant differences observed between substance use (cigarette/tobacco and alcohol) and gambling craving are consistent with the tendency of risk-related behaviors to co-occur. In addition, the finding that male participants reported higher levels of both gambling craving and deviant leisure tendencies suggests that risk-taking behaviors may vary across gender. Previous research has shown that men tend to have a higher tolerance for perceived risk in gambling contexts and may be more likely to engage in risky behaviors (6, 7). In this study, substance-related behaviors were operationalized as a broader behavioral orientation rather than isolated patterns, in order to reflect their co-occurring and risk-related nature.

The finding of a low but significant relationship between loneliness and gambling craving suggests that the association between loneliness and gambling may be more nuanced and context-dependent, rather than indicative of a strictly secondary role. The fact that loneliness does not strongly explain gambling craving directly may indicate that gambling among university students is experienced more within social and recreational contexts. However, the literature shows that loneliness is particularly associated with escape motivated gambling. While studies indicate that individuals experiencing loneliness tend to turn to online gambling communities (18, 20) it has also been demonstrated that loneliness may create a basis for problematic gambling over time (19). Similarly, recent research indicates that loneliness does not operate as an isolated predictor of gambling-related outcomes but rather reflects a more complex psychosocial process that unfolds in interaction with factors such as stress and individual vulnerability (21, 29). Although loneliness showed a weaker association with gambling craving, this should not be interpreted as a negligible role. Its contribution may be context-dependent and operate through indirect or conditional pathways, potentially in interaction with broader risk-oriented leisure tendencies. Therefore, loneliness may be better understood within a more integrative framework rather than as an independent predictor.

The finding that individuals who engage in leisure in a passive manner report higher levels of gambling craving and deviant leisure tendencies indicates a potential association with the quality of leisure. It has been suggested that unstructured and purposeless leisure may create a basis for risky behaviors among adolescents and young adults (12). At the same time boredom and sensation seeking have been shown to be associated with addiction like behaviors (11). In this context the content of leisure and the form of participation appear to be important factors. The increase in gambling craving as weekly leisure duration and screen time increase suggests that digital access may facilitate risky leisure practices. It has been reported that digitalization contributes to the spread of sports betting and online gambling among students (3, 4). This situation provides important evidence that gambling has become normalized as an ordinary part of everyday life.

However, the negative relationships found between academic achievement and both gambling and deviant leisure tendencies suggest that academic engagement may function as a potential protective factor. It has been reported that gambling craving is associated with declines in academic performance and other adjustment problems (8, 10). Individuals who are oriented toward academic goals may have more developed time management and self-regulation skills which may reduce the tendency to engage in negative and risky behaviors. In this context, academic achievement may indirectly function as a preventive threshold against risky leisure habits, even if it does not operate as a direct protective factor, as it appears to be negatively associated with such tendencies.

Overall, the findings of this study indicate that gambling craving among university students is strongly associated with deviant leisure tendencies, while loneliness appears to play a more limited but still meaningful role. These results suggest that gambling-related behaviors may be more appropriately understood within a broader leisure context, taking into account how leisure is structured, the social environments in which it occurs, and the underlying risk orientations involved. Accordingly, interventions may benefit from going beyond individual-level approaches and considering the potential role of structured recreational opportunities that promote active participation and social interaction. However, such implications should be interpreted cautiously, given the correlational nature of the study.

Although the study is conceptually informed by a broader perspective that includes social and contextual influences, the empirical design is limited to individual-level measures. Therefore, the findings should be interpreted within this scope, and future research is encouraged to incorporate relational and contextual variables to better align with the theoretical framework.

In conclusion, approaching gambling craving as part of a broader pattern of risk-oriented leisure engagement may offer a more comprehensive perspective. The findings related to group differences also align with the hypotheses, with large effect sizes observed for substance use and leisure type, suggesting that these factors are associated with gambling craving. These interpretations should be considered within a correlational framework and do not imply causal relationships.

### Theoretical implications

The following interpretations should be understood as conceptual reflections on observed associations rather than causal explanations. This study offers a theoretical contribution by extending beyond approaches that primarily explain gambling craving through individual psychopathology or impulse control disorders and by repositioning this behavior within the framework of deviant leisure. The findings suggest that deviant leisure tendencies emerge as a stronger statistical correlate of gambling craving, suggesting that gambling among university students may be interpreted not solely as an individual maladaptive behavior but also within the context of broader risk oriented leisure patterns.

The results of the study suggest that risky behaviors tend to co-occur within a particular lifestyle orientation. In this context gambling craving can be interpreted as part of a broader leisure orientation characterized by sensation seeking the stretching of normative boundaries and pleasure oriented consumption practices. Therefore, the study highlights that explanations of gambling should not be limited to individual deficits or psychological vulnerabilities but should also consider the leisure structures and cultural contexts within which individual behaviors are shaped.

In addition the relatively limited explanatory power of the loneliness variable suggests that psychosocial vulnerabilities alone may not be sufficient to explain gambling craving but may instead play a reinforcing role within certain risky leisure contexts. This finding indicates that emotional states may be linked to risk behaviors only under specific social and structural conditions and therefore gambling craving should be addressed within a multilayered theoretical framework. In this respect the study contributes to the development of an integrative conceptual perspective linking leisure theory the behavioral addiction literature and research on emerging adulthood.

However, it should be emphasized that these interpretations are based on correlational findings and do not imply causal relationships. Accordingly, the theoretical and practical implications derived from this study should be considered as preliminary and subject to further investigation using longitudinal or experimental research designs.

### Practical implications

The results suggest that patterns of leisure engagement may be associated with gambling craving among university students. In this context, the structuring of leisure and the development of recreational opportunities within university environments may help inform future research directions regarding potential interventions. In particular, programs that aims to channel students' search for excitement and stimulation into socially engaging and structured activities may represent a promising direction. However, the effectiveness of such approaches requires empirical testing through longitudinal or experimental research designs.

In addition, the observed associations between screen time and gambling craving suggest that digital environments may play a role in shaping these tendencies. Accordingly, initiatives focusing on digital awareness and risky online behaviors may be explored within future research contexts, rather than being interpreted as directly established intervention strategies. More broadly, the findings suggest that gambling craving may be associated with a wider pattern of risk-related behaviors. Therefore, future research may benefit from examining integrated approaches that consider substance use, leisure practices, and digital engagement together, particularly in the context of early identification of risk patterns among university students.

## Limitations and future research

This study provides important insights; however, several limitations should be considered when interpreting the findings. First, the cross-sectional design limits the ability to draw causal inferences regarding the relationships between variables. Future longitudinal studies may provide a clearer understanding of changes over time and the reciprocal dynamics between deviant leisure tendencies and gambling craving.

Second, the sample was limited to students from two universities, which restricts the generalizability of the findings. Research conducted with larger and more diverse samples across different sociocultural contexts, including cross-cultural comparisons, may strengthen the external validity of the results.

Third, the use of self-report measures raises the possibility of common method bias and social desirability effects. In addition, the conceptual proximity between deviant leisure tendencies and gambling craving suggests a potential overlap in measurement, which may partially account for the strength of the observed associations.

Data collection mode (online vs. face-to-face) was explicitly tested and statistically controlled within the hierarchical regression model. The results indicated that collection mode was not a significant predictor and did not meaningfully alter the pattern of findings. Accordingly, the main associations reported in this study do not appear to be driven by differences in data collection mode. Nevertheless, given that mixed-mode designs can introduce subtle variability in sensitive self-report measures, future research may benefit from more standardized data collection procedures.

Although the present study incorporated a range of demographic and behavioral variables as control factors within a hierarchical regression framework, other potentially relevant variables (e.g., psychological traits, social context, or environmental influences) were not included. Therefore, the findings should be interpreted within the scope of the variables examined.

Finally, the relatively limited role of loneliness in the current model suggests that this relationship may be influenced by additional mediating or moderating variables. Future studies are encouraged to develop more comprehensive theoretical models incorporating factors such as impulsivity, emotion regulation, social support, and psychological wellbeing.

The absence of direct measures of contextual and relational variables (e.g., perceived social support, family dynamics, and peer norms) represents another limitation, as these factors may play a significant role in shaping gambling-related behaviors. In this regard, future research may benefit from integrating both individual and contextual factors within a unified framework.

The use of advanced statistical techniques, such as structural equation modeling, may also allow a more detailed examination of the complex relationships between individual psychological characteristics and leisure structures.

## Data Availability

The raw data supporting the conclusions of this article will be made available by the authors, without undue reservation.
